# Evaluation of *in vitro* ruminal fermentation of ensiled fruit byproducts and their potential for feed use

**DOI:** 10.5713/ajas.18.0282

**Published:** 2018-05-31

**Authors:** Shimaa A Mousa, Pradeep K. Malik, Atul P. Kolte, Raghavendra Bhatta, Shigemitsu Kasuga, Yutaka Uyeno

**Affiliations:** 1 Graduate School of Science and Technology, Shinshu University, Minamiminowa 3994598, Japan; 2 Faculty of Veterinary Medicine, South Valley University, Qena 83523, Egypt; 3 Energy Metabolism Laboratory, ICAR-National Institute of Animal Nutrition and Physiology, Bengaluru 560030, India

**Keywords:** Fruit Byproducts, Ensiling, Methane, Tannins

## Abstract

**Objective:**

Ensiling of tannin-rich fruit byproducts (FB) involves quantitative and qualitative changes in the tannins, which would consequently change the rumen fermentation characteristics. This study aimed to evaluate whether ensiled FBs are effective in mitigating methane emission from ruminants by conducting *in vitro* assessments.

**Methods:**

Fruit byproducts (grape pomace, wild grape pomace, and persimmon skin) were collected and subjected to four-week ensiling by *Lactobacillus buchneri* inoculant. A defined feed component with or without FB samples (both fresh and ensiled material) were subjected to *in vitro* anaerobic culturing using rumen fluid sampled from beef cattle, and the fermentation parameters and microbial populations were monitored.

**Results:**

Reduced methane production and a proportional change in total volatile fatty acids (especially enhanced propionate proportion) was noted in bottles containing the FBs compared with that in the control (without FB). In addition, we found lower gene copy number of archaeal 16S rRNA and considerably higher levels of one of the major fibrolytic bacteria (*Fibrobacter succinogenes*) in the bottles containing FBs than in the control, particularly, when it was included in a forage-based feed. However, in the following cultivation experiment, we observed that FBs failed to exhibit a significant difference in methane production with or without polyethylene glycol, implying that tannins in the FBs may not be responsible for the mitigation of methane generation.

**Conclusion:**

The results of the *in vitro* cultivation experiments indicated that not only the composition but also ensiling of FBs affected rumen fermentation patterns and the degree of methane generation. This is primarily because of the compositional changes in the fibrous fraction during ensiling as well as the presence of readily fermented substrates, whereas tannins in these FBs seemed to have little effect on the ruminal fermentation kinetics.

## INTRODUCTION

Food byproducts have been considered as reasonable and economical feed resources. Fruit byproducts (FBs), in particular, apple pomace (AP) and grape pomace (GP), have been widely evaluated as feed material [1,2]. Wild grape (*Vitis coignetiae*, VP), which is regionally planted in Japan, also generates pomace after juicing. Persimmon skin (PS, generated during the preparation of dried persimmon), which is rich in soluble crude fiber such as pectin as well as soluble carbohydrates, also seems to be an attractive energy source in feeds [3,4]. Functional rumen microbial ecosystem is indispensable for rumen homeostasis and efficient carbohydrate conversion. In particular, methane production in ruminants has attracted considerable attention in relation to its contribution to greenhouse gas effect and global warming [5]. To date, although several means have been proposed for reducing methane emissions, innovative strategies that can substantially decrease methane output from livestock without compromising production have not yet been reported, indicating the need for elaborate assessment [6,7]. Tannins, which can form complexes with microbial proteins, are currently being intensively investigated in order to optimize rumen fermentation [8]. The potential benefits of feeding FBs that contain certain amounts of tannin in the inedible parts (seed, skin, and hull) might include manipulation of the rumen microbial community to reduce methane eructation and improve feeding efficiency. Since FBs are usually aqueous and their generation is usually limited to the harvest season every year, suitable measures need to be implemented to ensure their long-term preservation, such as lactic acid fermentation (ensiling). We previously conducted fermentation experiments of various FBs (AP, GP, and PS) to compare the fermentation quality and aerobic stability by using different lactic acid bacteria (LAB) and identified some LAB species that led to the production of high-quality FB silage [9]. In addition to decreasing soluble sugar content during silage fermentation, ensiling of tannin-rich FBs might involve quantitative and qualitative changes in the tannins [4,10], which would consequently change the rumen fermentation characteristics when the silage is fed to ruminants. This study aimed to evaluate whether ensiled tannin-rich FBs are effective in mitigating methane emission from ruminants by conducting *in vitro* assessments.

## MATERIALS AND METHODS

### Preparation of fruits byproduct silages

Three types of FBs (GP, VP, and PS) were obtained from different food processing factories in Nagano Prefecture, Japan. Grape pomaces were collected from different breweries after juicing of *Vitis labrusca* (Niagara, as GP) and *Vitis coignetiae* (as VP). Persimmon skin was collected from a processing plant for dried persimmon. We collected respective FB samples from each factory twice during each harvesting season in 2016, and all the FB samples were frozen immediately after collection until use. After thawing, two batches of equal amounts of each FB sample were mixed well, and proximate composition in the mixture was analyzed according to the official methods of AOAC [11]. The total extractable phenolics and condensed tannins (CTs) were determined using the method of Makkar [12]. The CT contents in the FBs were quantified as leucocyanidin equivalents as reported in previous studies [12]. The nutritional values of each FB are shown in Table 1. Before ensiling, the water content of the PS samples was adjusted by the addition of corn cobs to 95 g/kg dry matter (DM) PS. As in our previous study, we used *Lactobacillus buchneri* (*L. buchneri*) NBRC107764 for fermentation in this study [9,13]. Stock culture of this strain was used for inoculation of FB (1% v/w). The inoculated material was mixed well by hand, and 50 g of each was packed in a three-layer film bag. The bags were vacuum packed and tightly heat sealed (SQ-205S; Asahikasei Packs Co. Ltd., Tokyo, Japan), and then incubated at 25°C. Ensiled content was diluted by the addition of 180 mL of saline to the bags and incubated for 2 h at 5°C. The diluted sample was used to determine the pH, LAB counts (by using MRS agar [Oxoid, Basingstoke, UK]), yeast counts (by using chloramphenicol-added potato dextrose agar), and organic acids (by using HPLC as described previously [13]). The FBs ensiled for 28 days were then screened for their rumen fermentation modulation ability by using the *in vitro* culture method. All samples were air-dried at 60°C, ground, passed through a 1 mm screen, and stored at −20°C until they were used for the *in vitro* culture test.

### *In vitro* fermentation test

The method of incubation was the same as that used in our previous study [14]. Rumen fluid samples were collected from Japanese Black beef cows via a rumen fistula immediately before the morning feeding. Animal handling was performed according to the Shinshu University guidelines. The cows were fed Italian ryegrass straw and commercial concentrate at a 1:1 ratio. The collected rumen fluid was strained through four layers of cheesecloth and diluted (1:2) with pre-warmed McDougall buffer, which had been flushed with CO_2_ gas, and then used within 2 h after collection. The diluted rumen fluid (40 mL) was dispensed into a 100 mL serum bottle containing substrate (1.0 g), and then was flushed again with CO_2_ gas. Experimental substrates were prepared as follows: in Experiment 1, for each of the two types of basal feed mixture (concentrate-based and forage-based, including 80% of a designated part and 20% of the remaining part), fresh FBs that were maintained at −20°C (FRE), ensiled FB (SIL), or the basal feed (CONT) was included at one-third the level of the total DM. The concentrate was a commercial product and the forage was dried Italian ryegrass (Table 1). In Experiment 2, a forage-based feed was mixed with FRE, SIL, or CON to one third of total DM; subsequently, each feed mixture was subdivided into two, one of which included polyethylene glycol (PEG; 200 mg/g feed; PEG-6000; Wako Pure Chemical Industries Ltd., Osaka, Japan) as a tannin binder. The PEG was added to determine the effects of tannin on *in vitro* methanogenesis [2,15]. Because of material availability, Experiment 2 was performed using only GP and PS samples. The serum bottles (n = 3 per group) were sealed with a butyl rubber stopper and aluminum cap, and then incubated anaerobically for 24 h at 39°C with shaking at 180 rpm in a water bath.

### Sample analysis

After incubation, fermentation parameters (headspace gas composition, organic acid content, and methane generation) were analyzed according to the methods described previously [16,17]. The total bacterial numbers, methanogens, and fibrolytic bacteria were quantified using a real-time polymerase chain reaction method. Genomic DNA of the microorganisms was extracted using the QIAamp DNA Stool Mini Kit (Qiagen, Hilden, Germany) according to the manufacturer’s protocol, and the nucleic acid material was stored at less than −20°C until analysis. The polymerase chain reaction (PCR) conditions and primer sequences for total bacteria, Archaea, *Fibrobacter succinogenes*, and *Ruminococcus flavefaciens* were following a previous literature [18,19]. Primer sets for the real-time PCR were Eub338F (ACTCCTACGGGAGGCAG) and Eub522R (ACGTCRTCCMCNCCTTCCTC) for total bacteria, qmcrA-F (TTCGGTGGATCDCARAGRGC) and qmcrA-R (GBAR GTCGWAWCCGTAGAATCC) for Archaea, Fsuc3F (GTTC GGAATTACTGGGCGTAAA) and Fsuc3R (CCCCCGGAC ACCCAGTAT) for *F. succinogenes*, and RumFla3F (TGGCGG ACGGGTGAGTAA) and RumFla3R (TTACCATCCGTTTC CAGAAGCT) for *R. flavefaciens*. CFX96 Real-Time system (Bio-Rad Inc., Hercules, CA, USA) and a SYBR(R) Premix Ex Taq Kit (Takara Bio Inc., Otsu, Japan) were applied for the real-time PCR. The cycling conditions were initial denaturation at 95°C for 10 s, and 40 cycles at 95°C for 5 s, 62°C for 30 s, followed by melting curve analysis to confirm that expected PCR products were obtained.

### Statistical treatment

Analysis of variance (ANOVA) was applied for each measurement in each experiment. The following model was used:

$${\text{Y}}_{\text{ijk}}=\mu +\alpha_{\text{i}}+\beta_{\text{j}}+\gamma_{\text{k}}+{\left(\alpha \beta \right)}_{\text{ij}}+{\left(\beta \gamma \right)}_{\text{jk}}+{\left(\gamma \alpha \right)}_{\text{ki}}+{\left(\alpha \beta \gamma \right)}_{\text{ijk}}+{\text{e}}_{\text{ijk}}$$

Where Y_ijk_ = observations for dependent variables; μ = overall mean; α_i_ = the fixed effect of FB material (GP, VP, PS); β_j_ = the fixed effect of replacement (CONT, FRE, SIL); γ_k_ = the fixed effect of base-feed (CONC, FOR; Experiment 1) or PEG inclusion (Experiment 2); (αβ)_ij_, (βγ)_jk_, (γα)_ki_, (αβγ)_ijk_ = the interaction effect; and e_ijk_ = the residual error. Prior to ANOVA, our model assumptions were subjected to both a robust test for equality of variances, and Shapiro–Wilk’s test for normality of residual data to check validity of the model. A value of p< 0.05 on least-squares means was considered to indicate a significant effect of the treatment. Tukey’s pairwise comparison was applied for post-hoc test. All statistical analyses were performed with Stata 13.1 (Stata Corp, College Station, TX, USA).

## RESULTS AND DISCUSSION

### Quality of ensiled FBs

Developing tannin-rich FBs for feed use requires appropriate management to obtain high-quality silage without causing nutritional losses by aerobic deterioration. The fermentation data of the FBs are shown in Table 2. After 28 days of fermentation, the addition of LAB did not further decrease the pH compared to that in the control in GP and VP, and only lactate and acetate, but not propionate or butyrate, were detected in all the samples. However, the addition of LAB contributed to increased lactic acid fermentation in PS silage. *L. buchneri* is known to be effective in preventing aerobic deterioration when applied as an inoculant for ensiling plant feeds [20,21], and its use in silage preparation contributes to DM recovery [22]. We previously reported that the GP and PS ensiled with *L. buchneri* showed no temperature increase under anaerobic condition, indicating positive (i.e., preservative) effect in these silages [9]. Therefore, LAB inoculation of VP silage may have a positive effect on its storage stability even though yeast would survive in it. Indeed, we observed no aerobic deterioration for more than 10 days after opening the LAB-included VP silage, whereas slight (approximately 1°C) temperature increases were found in the raw material and control silage (data not shown).

### Effects of replacement with FBs on the *in vitro* rumen fermentation

In Experiment 1, we conducted an *in vitro* assessment to evaluate whether ensiled or non-ensiled FBs could be effective in modulating rumen fermentation as well as changing methanogen proportion (Table 3). As revealed by the volatile fatty acid (VFA) proportion, supplying any kind of FBs increased gas production and the changed the fermentation direction to propionate production as a hydrogen sink from the generation of methane. Moreover, total VFA was likely increased with the FB supplementation, but according to postestimation results of the interaction between fruits and base feed (Supplementary Table S1), the effect was only significant when PS was applied, presumably because of the higher soluble sugar concentration in PS. Interestingly, concurrent results were obtained in the increase of gas production and the decrease of acetate proportion in response to addition of either type of FB (fresh material or ensiled one) to concentrate-based feed, whereas these results were marginally observed only with the addition of fresh material to forage-based feed. Increase in gas production (i.e., fermentation intensity) in response to addition of fresh material is probably due to fermentable sugars in the FB. On the other hand, we speculate that there may be different microbes responsible for FB fermentation according to its status (fresh material or ensiled one) since different results were obtained with respect to the base feed. Methane production was significantly decreased in response to FB supplementation when it was added to forage-based feed. Interestingly, the proportion of archaea to total bacteria was the lowest in SIL, and significantly lower in FRE than in CONT. This was due to the increase in total bacterial number in SIL within forage-based feed rather than decreasing archaeal population. As compared with fresh FBs, the ensiled ones showed no effect on other measurements, except for marginal difference in NH_3_-N.

We hypothesized that the effects of replacement of feed with ensiled FBs on the *in vitro* rumen fermentation patterns were also probably because of the changes in the form of tannins during fermentation. FBs generally include non-edible parts of agricultural products, which often contain phenolic compounds such as tannins. The extractable phenolic compounds were marginally higher in PS than in GP, whereas CT levels were similar between the two FBs (Supplementary Table S3). This suggested that phenolic compounds other than CT, such as hydrolyzable tannin, were higher in PS than in GP, consistent with the findings of a previous study [23]. Antimethanogenic activities of tannins have been extensively revealed in several *in vitro* and *in vivo* studies [24,25], although not completely. We also evaluated the effects of six commercially available natural sources of tannins on the total archaea by conducting *in vitro* culture experiments and found that CTs reduced methane production by 5.5% and suppressed the population of methanogenic archaea by 12.0% [15]. The total archaeal population was lower when the combination of two types (hydrolyzed and CTs) was used than when hydrolyzed tannins were used alone, which might be attributed to the different modes of action of these kinds of tannins.

We assumed that, in addition to tannin, the characteristics of carbohydrate composition in these FBs might also primarily determine the fermentation patterns of the *in vitro* rumen culture. Compared to other plant materials that are rich in tannin, inclusion of highly digestible carbohydrates with a certain amount may be a particular nature of FBs. The NFC can be immediately digested by the major members of the rumen flora (e.g., *Bacteroidetes* and *Firmicutes*) [26], and anaerobic conversion into organic acids such as succinate, propionate, and butyrate can function as an alternative hydrogen sink to methane. Theoretically, since carbohydrates available for anaerobic digestion in the *in vitro* culture in ensiled FBs included a greater amount of fiber than that in fresh FBs, intensive fiber digestion by fiber-degrading bacteria might occur resulting in the increased production of acetate accompanied with hydrogen, which was used for the reduction of CO_2_ to methane generation. However, the amount of methane production was higher in FRE than in SIL. Conversely, as shown recently, digestibility can be improved by ensiling total mixed ration (TMR) in an *in vivo* digestibility assessment [27,28], which was possibly associated with enhanced fiber digestion in the rumen. We observed that ensiled TMR resulted in more methane production under *in vitro* rumen cultivation than fresh (non-ensiled) TMR [29]. Thus, ensiling of FBs might induce changes in the fiber composition, which would in turn offer favorable conditions for the growth of these fibrolytic bacteria.

Therefore, we performed additional *in vitro* cultivation experiment to evaluate in detail the relationship between the compositional changes in FBs during ensiling and the rumen fermentation characteristics and microbial profiles involved in fermentation (Experiment 2). In this experiment, we chose a forage-based feed component for the testing because it exhibited more prominent reduction than did the concentrate-based one in methane generation in response to FB addition. In accordance with the previous experiment, in Experiment 2 a marginal decrease in methane production was observed between the FRE and SIL groups (Table 4). Inclusion of ensiled FBs (SIL) changed the carbohydrate profiles of the test feeds during cultivation experiments compared to those in the control (CTL, no FB addition) and even to those of FRE (Supplementary Table S3). In SIL group, the fraction of non-fiber carbohydrates (NFC) was marginally lower than that in FRE. This suggested that bacteria contributing to silage fermentation (e.g., *Lactobacillus* species) utilized these readily available carbohydrates to convert to lactate and acetate. In contrast, with the inclusion of NDF, overall carbohydrate composition was different between GP and PS. These compositional changes seemed to certainly affect the microbial proportions during *in vitro* cultivation, especially for the two representative fibrolytic bacteria. We assumed that partially degraded fiber generated from ensiling provided more suitable fermentation substrates for *Fibrobacter* than for *Ruminococcus*, and the difference in the bacteria that participated in fiber degradation might have resulted in switching the fermentation product to limited hydrogen generation, which was expected to reduce methane production. This idea is partly supported by results obtained from Experiment 2, because the absolute number of *Fibrobacter* was higher in the PS group than in the GP group. However, determining fermentation kinetics in the rumen is seemingly insufficient owing to the monitoring of limited microbiota. A comprehensive assessment of the microbiota with the pyrosequencing approach will enable us to understand what is changing in the rumen in response to the intervention.

The results of Experiment 2 implied that mitigation of methane production could be partly attributed to tannins. However, because we observed that two kinds of FBs failed to exhibit a significant difference in any parameter with or without PEG, except for total gas production and the interaction of the replacement and PEG inclusion on acetate proportion, the addition of PEG may have alleviated the adverse proportion changes in FRE and SIL as compared with CONT. This observation might suggest that tannins in FBs may have considerable effect on changes in fermentation pattern in the rumen owing to some minor modifications of rumen microbe composition. However, it cannot be responsible for the mitigation of methane production as this effect seemed to depend largely on the carbohydrate profile of the FBs. Owing to the differences between hydrolyzed tannin and CT in terms of effects on fermentation in the rumen, determining the protein binding capacity of tannins present in FBs might be important for assessing the extent to which they would affect rumen microbes [30]. The finding that no significant effects of tannins on methane generation were observed may be mostly attributed to the limited amount of FB inclusion (one-third to total DM), which was aimed at practical implementation.

## CONCLUSION

The results of the *in vitro* cultivation experiments indicated that not only the composition but also ensiling of FBs affected rumen fermentation patterns and the degree of methane generation in *in vitro* culture. Our results suggested that ensiled FBs could initially have some direct or indirect effects on the reduction of methanogens. This is primarily because of the compositional changes in the fibrous fraction during ensiling as well as the presence of readily fermented substrates, whereas tannins in these FBs seemed to have little effect on the *in vitro* ruminal methane generation. Animal feeding experiments are warranted to determine whether feeding FBs increases feed efficiency owing to the improvement of fiber digestibility, or whether the effect might be offset by increasing methane emission. Detailed monitoring of the digestion kinetics of nutrients, as well as of the microbial interactions within the ecosystem by performing animal experiments might be needed for the practical application of ensiled FBs as feed for optimized rumen fermentation.

## Supplementary Data



## Figures and Tables

**Table 1:** Proximate composition of fruit byproducts (fresh matter) and feeds used for *in vitro* cultivation experiment

Items	GP	VP	PS	Concentrate	Italian ryegrass
DM (g/kg)	335	445	251	931	905
CP (g/kg DM)	95	145	45	180	95
NDF (g/kg DM)	250	340	220	220	680
NFC (g/kg DM)	495	448	735	405	85
TEPH (g/kg DM)	113	103	210	NA	NA
CT (g/kg DM)	71	59	85	NA	NA

GP, grape pomace; VP, wild grape pomace; PS, persimmon skin; DM, dry matter; CP, crude protein; NDF, neutral detergent fiber; NFC, non-fiber carbohydrates; TEPH, total extractable phenolics; NA, not available; CT, condensed tannins.

**Table 2:** Profiles of fruits byproducts and its silages tested in *in vitro* experiments

Item	GP		VP		PS	
		
	FRE	SIL	FRE	SIL	FRE	SIL
pH	3.86	3.89	3.55	3.46	6.04	3.54
LAB (log CFU/g)	5.15	6.86	5.75	6.21	5.69	7.86
Yeast (log CFU/g)	7.49	6.02	6.11	6.12	6.81	1.80
Lactate (g/kg FM)	15.0	14.7	14.7	21.7	6.3	38.7
Acetate (g/kg FM)	2.9	3.0	8.5	9.6	2.9	16.9

GP, grape pomace; VP, wild grape pomace; PS, persimmon skin; FRE, fresh material; SIL, silage; LAB, lactic acid bacteria; CFU, colony forming units; FM, fresh matter.

**Table 3:** *In vitro* rumen fermentation characteristics of concentrate-based or forage-based rations containing ensiled fruits byproducts

Item^2)^	Fruit			Base feed		Replacement			SE	Contrasts			Interaction			
				
	GP1) (18)	VP1) (18)	PS1) (18)	Concentrate1) (27)	Forage1) (27)	CONT1) (18)	FRE1) (18)	SIL1) (18)		Fruit (F)	Base feed (B)	Replacement (R)	F×B	F×R	B×R	F×B×R
Gas production (mL)	24.5	24.3	25.6	29.4	20.2	21.5	27.0	25.9	0.8	0.12	<0.01	<0.01	0.06	0.39	<0.01	0.07
Lactate (mmol/L)	1.3	1.2	0.7	0.9	1.1	1.2	0.9	0.9	0.1	0.08	0.45	0.22	0.25	0.35	0.40	0.24
Total VFA (mmol/L)	85.3	84.2	89.9	97.6	75.3	80.6^a^	93.4^b^	85.4^ab^	1.9	0.02	<0.01	<0.01	0.02	0.06	0.26	0.17
Acetate (mol %)	59.3	59.7	61.0	56.9	63.1	63.9	58.8	57.3	0.7	0.25	<0.01	<0.01	0.46	0.28	0.03	0.79
Propionate (mol %)	30.1	30.1	30.4	32.8	27.6	27.8^a^	31.2^b^	31.7^b^	0.6	0.93	<0.01	<0.01	0.37	0.67	0.15	0.96
Butyrate (mol %)	10.6	10.2	8.6	10.3	9.3	8.3^a^	10.0^ab^	11.1^b^	0.4	0.06	0.18	0.01	0.37	0.19	0.11	0.24
NH_3_-N (mmol/L)	38.6	39.4	38.0	39.8	37.5	38.0	39.6	38.4	0.3	<0.01	<0.01	<0.01	<0.01	0.60	<0.01	0.20
Methane (mmol/L)	12.6	13.0	13.1	11.9	13.8	14.9	12.6	11.0	0.3	0.30	<0.01	<0.01	0.15	0.51	<0.01	0.16
Total bacteria (log10 copies/mL)	8.0	8.0	8.0	8.0	8.0	7.9	7.9	8.1	0.0	0.31	0.46	<0.01	0.19	0.27	<0.01	0.40
Archaea (log10 copies/mL)	6.2	6.2	6.2	6.2	6.2	6.2	6.2	6.1	0.0	0.98	0.19	0.01	0.82	0.65	<0.01	0.12
Archaea (% total bacteria)	1.5	1.7	1.9	1.6	1.7	2.2^a^	1.8^b^	1.1^c^	0.1	0.10	0.23	<0.01	0.40	0.35	0.06	0.09

GP, grape pomace; VP, wild grape pomace; PS, persimmon skin, FRE, fresh material; SIL, silage; SE, standard error; VFA, volatile fatty acids.

1) Numbers in parenthesis indicate number of cultivation bottles in the experiment.

abc Values with different superscripts within the same row mean significant difference. When the interaction was significant, Tukey’s pairwise comparison was applied for each combination as a postestimation. Results of pairwise comparisons were shown in Supplementary Table S1 and S2.

**Table 4:** *In vitro* rumen fermentation characteristics of test feeds containing ensiled fruits byproducts and PEG1)

Item2)	Fruit		Replacement			PEG		SE	Contrasts			Interaction			
				
	GP1) (18)	PS1) (18)	CONT1) (12)	FRE1) (12)	SIL1) (12)	−PEG1) (18)	+PEG1) (18)		Fruit (F)	Replacement (R)	PEG (P)	F×R	F×P	R×P	F×R×P
Gas production (mL)	22.6	23.0	23.4	22.2	22.8	21.8	23.8	0.4	0.51	0.36	<0.01	0.99	0.47	0.24	0.14
Total VFA (mmol/L)	92.8	95.8	97.9	94.0	91.0	93.9	94.7	1.2	0.25	0.11	0.76	0.92	0.97	0.38	0.93
Acetate (mol %)	52.8	50.5	55.3	50.5	49.2	51.3	52.0	0.9	0.11	<0.01	0.21	0.50	0.93	<0.012)	0.55
Propionate (mol %)	34.6	35.0	33.3	35.5	35.6	34.9	34.7	0.5	0.83	0.16	0.73	0.38	0.70	0.07	0.57
Butyrate (mol %)	12.6	14.5	11.4^a^	14.1^b^	15.3^b^	13.8	13.3	0.6	0.07	0.01	0.18	0.80	0.81	0.07	0.76
NH_3_-N (mmol/L)	38.6	40.6	39.9	37.5	41.3	39.5	39.6	0.6	0.13	0.06	0.91	0.69	0.51	0.28	0.88
Methane (mmol/L)	13.1	13.8	15.1^a^	13.2^b^	12.1^c^	13.6	13.4	0.2	0.14	<0.01	0.32	0.84	0.13	0.57	0.62
Total bacteria (log10 copies/mL)	8.1	8.1	8.0	8.1	8.2	8.1	8.2	0.0	0.94	0.08	0.52	0.77	0.83	0.42	0.87
Archaea (log10 copies/mL)	6.2	6.1	6.2	6.1	6.1	6.1	6.2	0.0	0.73	0.33	0.20	0.68	0.95	0.99	0.91
Archaea (% total bacteria)	1.3	1.4	1.8^a^	1.4^ab^	0.9^b^	1.5	1.2	0.1	0.60	0.03	0.40	0.77	0.35	0.17	0.93
*Fibrobacter* (log10 copies/mL)	6.4	6.6	6.4^a^	6.5^a^	6.7^b^	6.5	6.5	0.0	0.04	<0.01	0.93	0.10	0.63	0.54	0.09
*R. flavefaciens* (log10 copies/mL)	5.3	5.4	5.4	5.3	5.3	5.4	5.3	0.0	0.44	0.67	0.18	0.59	0.96	0.12	0.59

PEG, polyethylene glycol; GP, grape pomace; PS, persimmon skin; CONT, basal feed; FRE, fresh material; SIL, silage; −PEG, without polyethylene glycol; +PEG, with polyethylene glycol; SE, standard error; VFA, volatile fatty acids.

1) Numbers in parenthesis indicate number of cultivation bottles in the experiment.

2) The result of postestimation showed that the following pairs were significantly different (p<0.01): (CONT−PEG) vs (FRE−PEG), (CONT−PEG) vs (SIL−PEG), (CONT−PEG) vs (SIL+PEG), (CONT+PEG) vs (FRE−PEG), and (FRE−PEG) vs (FRE+PEG).

abc Values within the row with different superscripts are significantly different (p<0.05).
