# Supplementary material for: Evaluation of in vitro ruminal fermentation of ensiled fruit byproducts and their potential for feed use
Source: Asian-Australas J Anim Sci. 2018 May 31;32(1):103–9. doi: 10.5713/ajas.18.0282 (PMC6325409; doi:10.5713/ajas.18.0282)
Supplement: Supplementary file 1 [file ajas-18-0282-supplmentary.pdf]

Supplementary Table S1. Pairwise comparisons of marginal linear predictions as postestimation of data in Experiment 1 (after ANOVA, regarding the interaction between base feed and replacement) [1)]

	Gas production	Acetate	NH_3-N	Methane	Total bacteria	Archaea
CC vs CR	**	*	*			**
CC vs CS	**	**		**		**
CC vs FC	**	*		**	**	**
CC vs FR	*					*
CC vs FS	*		**	*		*
CR vs CS			*	*		
CR vs FC	**	**		**		
CR vs FR	**	*	*			
CR vs FS	**	*	*		**	
CS vs FC	**	**		**	**	
CS vs FR	**	**		**		
CS vs FS	**	**	**			
FC vs FR	*	*		**		
FC vs FS			**	**	**	
FR vs FS			**	*	**	

[1)] Abbreviations: CC, concentrate and control; CF, concentrate and fresh material; CS, concentrate and silage; FC, forage and control; FF, forage and fresh material; FS, forage and silage.

Supplementary Table S2. Pairwise comparisons of marginal linear predictions as postestimation of data in Experiment 1 (after ANOVA, regarding the interaction between fruits and base feed) [1)]

	Total VFA production	NH_3-N
GC vs GF	**	*
GC vs VC		
GC vs VF	**	
GC vs PC	*	
GC vs PF	**	**
GF vs VC	**	*
GF vs VF		
GF vs PC	**	*
GF vs PF		*
VC vs VF	**	
VC vs PC	*	
VC vs PF	**	**
VF vs PC	**	
VF vs PF		**
PC vs PF	*	**

[1)] Abbreviations: GC, grape pomace and concentrate; GF, grape pomace and forage; VC, wild grape pomace and concentrate; GF, wild grape pomace and forage; PC, persimmon skin and concentrate; PF, persimmon skin and forage;.

Supplementary Table S3. Proximate composition of test feeds containing ensiled fruit byproducts in Experiment 2 [1)]

Fruit Replacement	CONT	GP		PS	
		FRE	SIL	FRE	SIL
DM (g/kg)	925	918	916	908	903
CP (g/kg DM)	120	118	115	105	106
NDF (g/kg DM)	550	567	553	441	433
NFC (g/kg DM)	145	180	164	313	272
TEPH (g/kg DM)	10	55	35	76	71
CT (g/kg DM)	2	26	30	29	30

1) GP, grape pomace; PS, persimmon skin, CP, crude protein; NDF, neutral detergent fiber; NFC, non-fiber carbohydrates; TEPH, total extractable phenolics; CT, condensed tannins. Cont means no inclusion of FB, FRE and SIL mean inclusion of corresponding materials of each FB.
